# RACK1 overexpression is linked to acquired imatinib resistance in gastrointestinal stromal tumor

**DOI:** 10.18632/oncotarget.7426

**Published:** 2016-02-16

**Authors:** Xiaodong Gao, Anwei Xue, Yong Fang, Ping Shu, Jiaqian Ling, Yingyong Hou, Kuntang Shen, Jing Qin, Yihong Sun, Xinyu Qin

**Affiliations:** ^1^ Department of General Surgery, Zhongshan Hospital, Fudan University, Shanghai, China; ^2^ Institute of General Surgery, Fudan University, Shanghai, China; ^3^ Department of Pathology, Zhongshan Hospital, Fudan University, Shanghai, China

**Keywords:** gastrointestinal stromal tumor, KIT, RACK1, imatinib

## Abstract

Although treatment with imatinib, which inhibits KIT and PDGFR, controls advanced disease in about 80% of gastrointestinal stromal tumor (GIST) patients, resistance to imatinib often develops. RACK1 (Receptor for Activated C Kinase 1) is a ribosomal protein that contributes to tumor progression by affecting proliferation, apoptosis, angiogenesis, and migration. Here, we found that c-KIT binds to RACK1 and increases proteasome-mediated RACK1 degradation. Imatinib treatment inhibits c-KIT activity and prevents RACK1 degradation, and RACK1 is upregulated in imatinib-resistant GIST cells compared to non-resistant parental cells. Moreover, Erk and Akt signaling were reactivated by imatinib in resistant GIST cells. RACK1 functioned as a scaffold protein and mediated Erk and Akt reactivation after imatinib treatment, thereby promoting GIST cell survival even in the presence of imatinib. Combined inhibition of KIT and RACK1 inhibited growth in imatinib-resistant GIST cell lines and reduced tumor relapse in GIST xenografts. These findings provide new insight into the role of RACK1 in imatinib resistance in GIST.

## INTRODUCTION

Gastrointestinal stromal tumor (GIST) is the most common sarcoma of the gastrointestinal tract [1, 2]. Most GIST cells aberrantly express c-KIT protein, a class III receptor tyrosine kinase that is stimulated by its ligand, stem cell factor. Activated c-KIT initiates a downstream signaling cascade including STAT, ERK1/2, and protein kinase B/Akt [3]. Imatinib mesylate (Gleevec), a tyrosine kinase inhibitor with activity against ABL1, ABL2, KIT, platelet-derived growth factor receptor α (PDGFRα), and PDGFRβ, is an effective treatment for GIST [4–6]. Although most GIST patients respond well to imatinib, acquired resistance to imatinib does occur in some patients, and acquired resistance is a major problem for targeted therapies in general. Patients who experience acquired resistance respond to imatinib treatment initially, but experience progression after 6 months of therapy. An important mechanism for acquired resistance to imatinib is reactivation of KIT, which occurs *via* secondary gene mutations in the KIT kinase domain [7–10]. Non-genetic acquired resistance mechanisms have also been reported. Javidi-Sharifi et al. showed that signaling crosstalk between KIT and FGFR3 promoted imatinib resistance in GIST [11]. Interestingly, viable GIST cells can be found in patients who undergo tumor resections during imatinib therapy [12], suggesting that residual GIST cells may adapt to the drug through the activation of other pathways.

The receptor for activated C-kinase 1 (RACK1) is a member of the tryptophan-aspartate repeat (WD-repeat) family of proteins [13]. RACK1 serves as a scaffold protein for many kinases and receptors and plays a pivotal role in a wide range of biological responses, including signal transduction, immune response, and cell growth, migration, and differentiation [14]. RACK1 is upregulated in several kinds of tumors and is considered an excellent marker of oral squamous carcinoma, breast cancer, and pulmonary adenocarcinomas [15–19]. Aberrant RACK1 expression contributed to *in vitro* chemoresistance in hepatocellular carcinoma. These effects depended on the association between RACK1 and ribosomes. Ribosomal RACK1 coupled with PKCβII to promote the phosphorylation of eukaryotic initiation factor 4E (eIF4E), which led to preferential translation of potent cell survival factors [20]. In the current study, we demonstrate that RACK1 plays an important role in the regulation of imatinib resistance in GISTs. Constitutively active c-KIT associated with RACK1 and decreased RACK1 stability by promoting its ubiquitin-proteasome degradation. Inhibiting c-KIT activity with imatinib increased RACK1 expression, and RACK1 reactivated signaling molecules downstream of c-KIT to promote imatinib resistance in GISTs. Future studies targeting RACK1 may lead to novel approaches that inhibit or reverse the development of imatinib resistance in GISTs.

## RESULTS

### RACK1 protein is overexpressed in imatinib-resistant GIST cells

**Figure 1:**
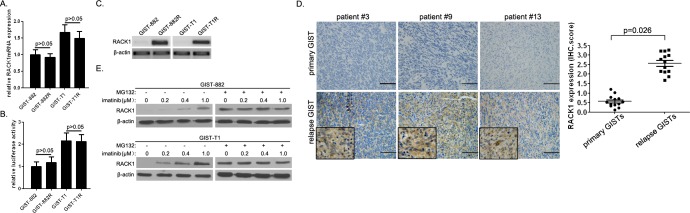
RACK1 is overexpressed in imatinib-resistant GIST cells **A.** RACK1 mRNA expression in GIST-882R, GIST-T1R, and parental imatinib-sensitive cells was assessed by real-time PCR. **B.** Relative promoter activity of pGL3-GNB2L1-luc in GIST-882R, GIST-T1R, and parental imatinib-sensitive cells. **C.** RACK1 protein levels in GIST-882R, GIST-T1R, and parental imatinib-sensitive cells assessed by Western blots. **D.** Immunohistochemical analysis of RACK1 in tumor sections taken from representative GIST patients before (primary tumors) and after (relapse tumors) imatinib treatment (left panels). Scale bars: 50 μm. RACK1 expression in relapse GISTs after imatinib treatment was compared to matched primary tumors (right panel; *n* = 12). E. RACK1 protein levels in imatinib-resistant sublines of GIST-882 and GIST-T1 cells with or without MG132 treatment were determined by immunoblotting. β-actin was used as an internal control. Bars represent the mean of triplicate samples; error bars represent standard deviation. Data are representative of three independent experiments.

In the current study, we established 2 cell line models of acquired resistance following continuous *in vitro* exposure to imatinib using GIST-882 and GIST-T1 cells. We compared RACK1 expression in imatinib-resistant cells and their parental counterparts using qPCR and Western blot analysis. RACK1 mRNA levels did not differ between imatinib-resistant cells and parental cells (Figure 1A). In line with this, the promoter construct pGL3-GNB2L1, which contains NF-κB elements essential for RACK1 transcription, showed transcriptional activity in both imatinib-resistant cells and parental cells (Figure 1B). However, RACK1 protein expression was higher in GIST-882R and GIST-T1R cells than in imatinib-sensitive clones (Figure 1C). To establish the clinical relevance of RACK1 expression in imatinib resistance, we assessed RACK1 expression in 13 GIST patients who had paired tumor specimens available from before and after imatinib treatment (primary *vs.* relapsed lesions). Representative sections showing RACK1 staining and comparisons of RACK1 expression between primary and relapse specimens are shown in Figure 1D. Although the morphology of relapse GISTs after imatinib treatment did not differ markedly from native tumors, all patients showed upregulated RACK1 protein expression in relapse lesions. However, RACK1 mRNA levels did not differ between primary and relapse lesions (data not shown).

Next, we evaluated RACK1 expression in the imatinib-resistant GIST-882 and GIST-T1 cell variants cultured continuously in gradually increasing doses of imatinib up to 1μM. When compared to their parental lines, the variants were 10- to 200-fold more resistant to imatinib (data not shown). We found that RACK1 protein, but not mRNA, expression was closely correlated with the degree of imatinib resistance (Figure 1E). More importantly, MG132, the proteasome inhibitor, equalized RACK1 levels in imatinib-resistant variants to those in parental cells (Figure 1E). These data suggest that RACK1 is overexpressed in imatinib-resistant GIST cells and that imatinib regulates RACK1 levels by inhibiting proteasomal degradation.

### Involvement of RACK1 in imatinib resistance of GIST cells

**Figure 2:**
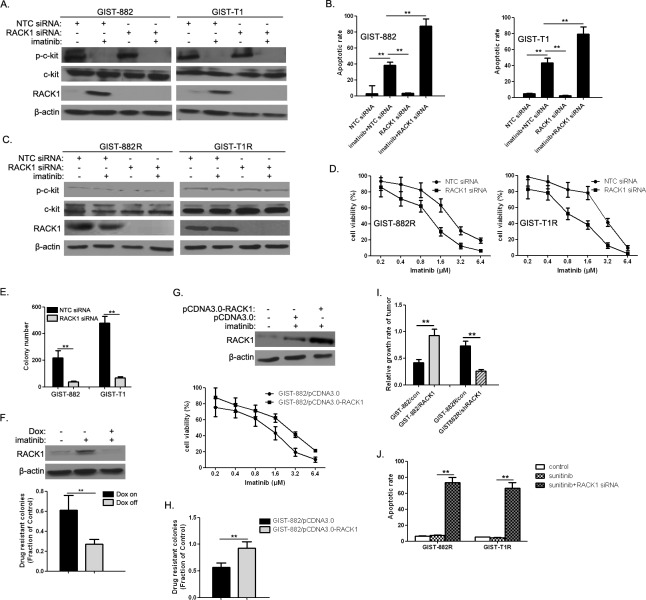
RACK1 modulates imatinib resistance **A.** After GIST-882 and GIST-T1 cells were treated with imatinib and/or RACK1 siRNA for 72h, p-KIT, total KIT, and RACK1 protein levels were determined by Western blot. **B.** The apoptotic rate was assessed by flow cytometry of Annexin V-FITC/PI staining (lower panel). **C.** After GIST-882 and GIST-T1 cells were treated with imatinib and/or RACK1 siRNA, p-KIT, total KIT, and RACK1 protein levels were determined by Western blot. **D.** Cell viability was assessed using Cell Titer-Glo assays. **E.** GIST-882 and GIST-T1 cells were transfected with either NTC siRNA or RACK1 siRNAs for 48 hr followed by imatinib (1μM) for 4 weeks. The number of imatinib-resistant colonies formed was quantified. **F.** Doxycycline-inducible shRACK1-expressing cells were established from GIST-T1 cells. RACK1 expression was detected by Western blot (upper panel). The transfectants were treated with imatinib (1μM) for 4 weeks with or without doxycycline to assess drug-resistant colony formation. The fraction of control colonies that developed imatinib resistance was quantified (lower panel). **G.** Generation of GIST-882 cells stably expressing pCDNA3.0 or pCDNA3.0-RACK1. RACK1 expression was detected by Western blot (upper panel). The transfectants were treated with imatinib for 72 hr to measure cell viability using Cell Titer-Glo assays (lower panel). **H.** The transfectants in (E) were treated with imatinib (1μM) for 4 weeks to measure drug-resistant colony formation. The fraction of control colonies that developed imatinib resistance was quantified. **I.** After *in vivo* imatinib treatment for 21 days, implanted tumor length and width were measured and relative growth rate was determined. **J.** GIST-882R and GIST-T1R cells were treated with 10 μM sunitinib either alone or in combination with RACK1 siRNA for 72 hours, after which the apoptotic rate was assessed by flow cytometry of Annexin V-FITC/PI staining. Bars represent the mean of triplicate samples; error bars represent standard deviation. Data are representative of three independent experiments. **p < 0.05 versus corresponding controls.

We examined whether RACK1 expression affected responses to imatinib in GIST cells. RACK1 depletion by RNAi (Figure 2A) in GIST-882 and GIST-T1 cells accelerated imatinib-induced apoptosis (Figure 2B). Moreover, RACK1 RNAi (Figure 2C) reduced viability in imatinib-treated GIST-882R and GIST-T1R cells (Figure 2D). To further explore the role of RACK1 in the development of acquired drug resistance, we treated RACK1 siRNA-transfected GIST-882 and GIST-T1 cells with imatinib for 4 weeks. RACK1 knockdown suppressed imatinib-resistant colony formation in both cell lines (Figure 2E), suggesting that early increases in RACK1 contribute to the development of drug resistance. To confirm this observation, we generated GIST-T1 cells expressing RACK1 shRNA under the control of a doxycycline (Dox)-dependent promoter and tested them in a colony formation assay. Inducible RACK1 knockdown (Figure 2F, upper panel) prevented the formation imatinib-resistant GIST-T1 colonies (Figure 2F, lower panel). We also generated GIST-882 cells that stably expressed RACK1 to examine its role in acquired drug resistance. As expected, ectopic RACK1 expression (Figure 2G, upper panel) induced a shift in the half-maximal inhibitory concentration (IC50) for imatinib in a 3-day viability assay (Figure 2G, lower panel) and dramatically increased the number of imatinib-resistant colonies (Figure 2H). We then determined the *in vivo* reactivity of RACK1-related transfectants to imatinib. Figure 2I shows that imatinib treatment strongly inhibited tumor growth in GIST-882-con and GIST-882R/shRACK1 tumors *(p <* 0.05), suggesting that mice receiving implantations of GIST-882-RACK1 and GIST-882R-con were more resistant to imatinib than those receiving GIST-882-con and GIST-882R/shRACK1, respectively. Interestingly, the imatinib-resistant variants were cross-resistant to another c-Kit inhibitor, Sunitinib (Figure 2J). These effects were largely reversed by RACK1 depletion (Figure 2J), indicating that RACK1 is also responsible for sunitinib resistance in this context.

### Activated c-KIT promoted ubiquitin-proteasome degradation of RACK1

**Figure 3:**
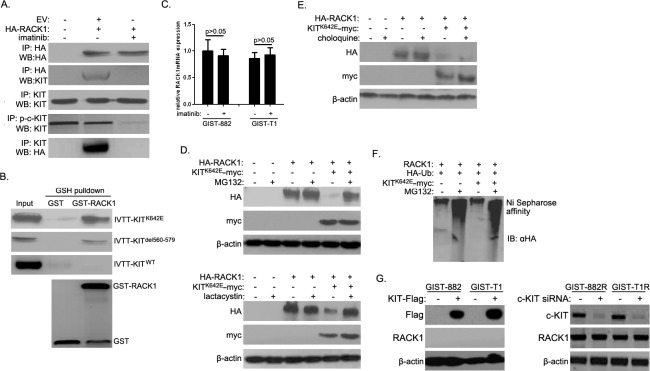
Activated c-KIT binds to RACK1 and is required for ubiquitin-proteasome degradation of RACK1 **A.** After 48 h of imatinib treatment, whole cell lysates were prepared from GIST-T1 cells transfected with empty vector (EV) or HA-RACK1. Immunoprecipitation (IP) was performed using anti-HA, anti-p-c-KIT, or anti-KIT antibodies. Immunoblotting assays were used to detect HA-RACK1, p-c-KIT and c-KIT in whole cell lysates and IP products. **B.** Purified GST or GST-RACK1 was incubated with *in vitro* transcribed and translated wild-type, K642E, or del580-570 mutants of c-KIT, captured on glutathione (GSH)-Sepharose beads, and analyzed by SDS-PAGE followed by autoradiography and an immunoblotting assay with anti-GST. **C.** RACK1 mRNA levels were assessed by quantitative PCR in GIST-882 and GIST-T1 cells with or without 24 h of imatinib treatment. **D.** HEK293 cells were transfected with pcDNA3.0/RACK1-HA with or without pcDNA3.0/KIT^K642E^-myc. 36 h after transfection, cells were treated with MG132 (10μM; upper panels) or lactacystine (10μM; lower panels) for another 5 h. Cell lysates were then subjected to Western blot using anti-HA or anti-myc antibody. **E.** HEK293 cells were transfected as in (D). 24 h after transfection, cells were treated with choloquine (10mM) for another 12 h. Cells lysates were then analyzed as in (D). **F.** HEK293 cells were transfected with pcDNA3.0/RACK1, pcDNA3.0/KIT^K642E^-myc, and pcDNA3/HA-ubiquitin constructs. 36 h after transfection, cells were treated with 10μM MG132 for another 5 h. Cell lysates were subjected to nickel agarose purification. The affinity-precipitated complexes were separated and blotted with anti-HA antibody. **G.** pCDNA3.0-KIT-Flag was transfected into GIST882 and GIST-T1 cells, while c-KIT siRNA was transfected into GIST882R and GIST-T1R cells. 48 h after transfection, cell lysates were subjected to Western blot using anti-Flag, anti-c-KIT, or anti-RACK1 antibodies.

Imatinib is a potent inhibitor of the tyrosine kinases c-KIT and PDGFR-α in GISTs [6]. Because imatinib markedly increased RACK1 expression, we investigated the interactions between RACK1 and c-KIT and PDGFR-α. Intriguingly, HA-RACK1 co-immunoprecipitated with endogenous c-KIT in GIST-882 and GIST-T1 cells (Figure 3A), and imatinib largely abolished this effect. However, there was no direct association between RACK1 and endogenous PDGFR-α. Purified GST-RACK1 bound to active c-KIT mutants (KIT^K642E^ in GIST-882 cells and KIT^del560-579^ in GIST-T1 cells), but not to wild-type c-KIT (Figure 3B), *in vitro*. These data suggest that activated c-KIT interacts with RACK1 in GIST cells.

Previous reports indicate that the c-KIT signaling pathway in melanocytes targets the transcription factor Mi and promotes ubiquitin-proteasome degradation [21]. Because inhibiting c-KIT activity with imatinib increased RACK1 protein levels, we speculated that activated c-KIT promotes RACK1 degradation *via* the ubiquitin-proteasome pathway. We first examined whether inhibiting c-KIT activity with imatinib modulates RACK1 mRNA levels using qPCR. Imatinib treatment had no effect on RACK1 mRNA levels (Figure 3C). Next, two proteasome inhibitors, MG132 and lactacystine, were used to suppress proteasome degradation. As shown in Figure 3D, overexpression of the constitutively active KIT^K642E^ mutant in HEK293T cells strongly down-regulated RACK1 protein levels, and treatment with proteasome inhibitors blocked RACK1 degradation. We also examined whether activated c-KIT promoted RACK1 degradation *via* the lysosome pathway. Treatment with choloquine, a lysosome inhibitor, did not affect RACK1 protein levels (Figure 3E). We also determined whether active c-KIT facilitated the degradation of RACK1 by enhancing its ubiquitination, which is a crucial step for proteasome-mediated degradation. Treatment with MG132 increased RACK1 ubiquitination, and overexpression of the KIT^K642E^ mutant increased ubiquitination even further (Figure 3F, lane1 *vs.* lane2, lane1 *vs.* lane4). However, KIT^K642E^ mutant overexpression did not increase RACK1 ubiquitination in the absence of MG132 (Figure 3F, lane 1 *vs.* lane 3), possibly due to the relatively low baseline RACK1 expression and accelerated c-KIT-mediated degradation (Figure 3D). We then overexpressed c-KIT in GIST882 and GIST-T1 cells and silenced c-KIT expression in GIST882R and GIST-T1R cells. As shown in Figure 3G, RACK1 levels did not change after either up-regulation or down-regulation of c-KIT, suggesting that imatinib does not regulate RACK1 protein expression though c-KIT. Collectively, these results demonstrate that active c-KIT down-regulates RACK1 protein levels by promoting RACK1 ubiquitination and subsequent proteasome-mediated degradation.

### RACK1 reactivates Ras/ERK and PI3K/Akt signaling in imatinib-resistant GIST cells

**Figure 4:**
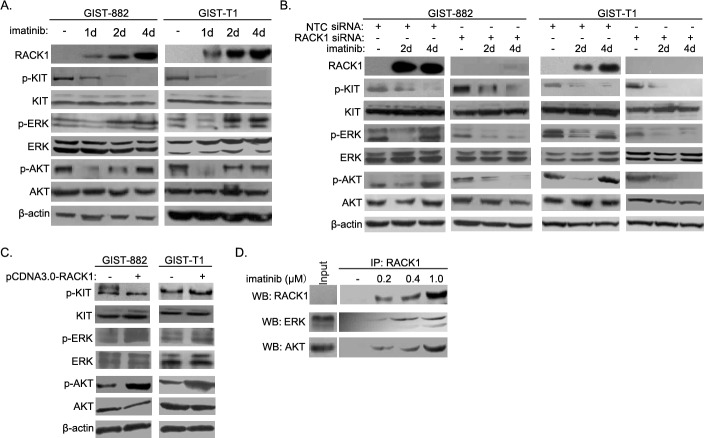
RACK1 reactivates the ERK and Akt pathways during induction of imatinib resistance in GIST cells **A.** GIST-882 and GIST-T1 cells were harvested after imatinib treatment for the indicated times, and whole cell lysates were analyzed by Western blotting for RACK1, p-c-KIT, total c-KIT, p-ERK, total ERK, p-AKT, and total AKT. **B.** GIST-T1 and GIST-882 cells were transfected with control or RACK1 siRNAs. Transfected cells were cultured for 72 hours and harvested after imatinib treatment for 2 or 4 days. Whole cell lysates were subjected to Western blot to determine the protein levels of RACK1, p-c-KIT, total c-KIT, p-ERK, total ERK, p-AKT, and total AKT. **C.** GIST-T1 and GIST-882 cells were transfected with pcDNA3.0/RACK1 or control vector, after which whole cell lysates were analyzed by Western blotting for p-c-KIT, total c-KIT, p-ERK, total ERK, p-AKT, and total AKT. **D.** Cell lysates from imatinib-resistant sublines of GIST-882 cells were subjected to immunoprecipitation with anti-RACK1 antibody, followed by Western blot with related antibodies as indicated.

Reactivation of downstream signaling contributes to acquired resistance to oncogene-targeting therapies. To identify the molecular mechanism underlying RACK1-mediated imatinib resistance, we assessed the activation of signaling molecules downstream of c-KIT in both short- and long-term GIST882 and GIST-T1 cell cultures. Treatment with imatinib strongly reduced c-KIT phosphorylation at tyrosine 721 (Figure 4A) in both cell lines, indicating that c-KIT activity was suppressed. After initial inhibition of ERK and Akt phosphorylation in both cell lines, imatinib treatments greatly increased ERK and Akt phosphorylation (Figure 4A). No similar phenomena were observed with other signaling molecules downstream of c-KIT, such as PLC-γ, Src, or JAK/STAT (data not shown). Of note, RACK1 knockdown in imatinib-treated GIST-T1 and GIST882 cells markedly inhibited reactivation of the ERK and Akt pathways (Figure 4B). On the contrary, overexpression of RACK1 in GIST-T1 and GIST882 cells further increased phosphorylation of ERK and Akt without affecting c-KIT activity (Figure 4C).

RACK1 is a classical scaffold protein which interacts with many proteins and regulates multiple signaling pathways [14]. We next examined the interaction between RACK1 and signaling molecules downstream of c-KIT. As shown in Figure 4D, RACK1 associated with ERK and Akt, and these interactions increased during the induction of imatinib resistance. However, no interactions were detected between RACK1 and PLC-γ, Src, or JAK/STAT (data not shown). Together, these results suggest that RACK1 functions as a critical scaffold protein in feedback reactivation of signaling molecules downstream of c-KIT after imatinib treatment.

### Imatinib in combination with RACK1 shRNA prevented GIST recurrence *in vivo*

**Figure 5:**
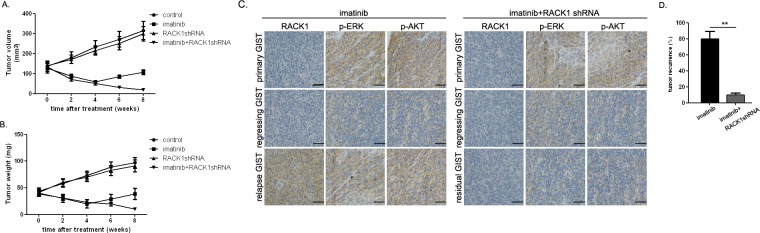
Combined imatinib and RACK1 shRNA treatment prevents GIST recurrence in xenografted mice GIST-882 cells transfected with RACK1 shRNA or control shRNA were subcutaneously implanted. When tumors reached an average volume of 150mm^3^, mice (20 per group) were treated with imatinib (250mg/kg, twice daily) or 0.1ml normal saline (pH 7.4, twice daily) for 4 days. Tumor volumes **A.** and weights **B.** were measured weekly after inoculation with imatinib or saline. **C.** RACK1-, p-Erk-, and p-Akt-stained sections of primary, regressing, or relapse/residual tumors from mice receiving imatinib alone or in combination with RACK1 shRNA. **D.** Mice were monitored weekly for local and distant recurrence. Bars represent the mean of triplicate samples; error bars represent standard deviation. Data are representative of three independent experiments. **p < 0.05 versus corresponding controls. Scale bars: 50μm.

Because RACK1 activated signaling molecules downstream of c-KIT, we examined whether enhanced antitumor activity occurred when imatinib and RACK1 shRNA were administered in combination. After 14 days, when established GIST-882 subcutaneous tumor xenografts were detectable, mice were treated for 8 weeks with imatinib with or without RACK1 shRNA. As shown in Figure 5A and 5B, treatment with imatinib alone produced > 50% tumor regression at week 4. However, tumors recurred in 8 out of 10 of these mice (Figure 5D). In these cases, RACK1 expression was remarkably upregulated in the relapsed GISTs as compared to the corresponding primary and regressing tumors, and Erk and Akt signaling were also reactivated (Figure 5C). RACK1 shRNA treatment alone did not affect tumor burden (Figure 5A and Figure 5B). In contrast, treatment with RACK1 shRNA plus imatinib decreased the size and the weight of tumors and abolished re-activation of Erk and Akt (Figure 5A and 5B). Thus, combined treatment reduced tumor recurrence, which was detected in only 1 of 10 mice (Figure 5D).

## DISCUSSION

Although most GIST patients respond well to imatinib, almost all of them develop resistance after long periods of treatment [8, 9]. Elucidating the mechanisms of resistance to oncogene-targeting drugs is critical for the development of more effective therapies. An extensive effort has been made to understand resistance to c-KIT inhibitors in GIST. Previous studies show that kinases activated by oncogenic mutations reprogram signaling networks and induce feedback inhibition of other pathways in cancer cells. Inhibitors selective for these kinases may relieve feedback inhibition and activate many additional pathways. Positive responses to these targeting therapies occur relatively quickly, and their effectiveness then diminishes [22–26]. The c-KIT molecule consists of a long extracellular domain, a transmembrane segment, and an intracellular part. Mutations generally occur in the DNA encoding the intracellular part, which acts as a tyrosine kinase and activates other enzymes. Upon activation, signaling proteins are recruited to c-KIT by certain interaction domains (e.g., SH2 and PTB) that specifically bind to phosphorylated tyrosine residues in the intracellular region of c-KIT, and then contribute to the development and malignant progression of GISTs [27, 28]. Here, we report a novel interaction between RACK1 and constitutively active c-KIT. This interaction was verified both *in vivo* and *in vitro* by co-immunoprecipitation and GST pull-down assays, respectively. Furthermore, c-KIT decreased the stability of RACK1 by promoting its ubiquitin-proteasome degradation. Inhibiting c-KIT activity with longer-term imatinib treatments promoted RACK1 expression *via* disruptions in the ubiquitin-proteasome pathway, and RACK1 was highly expressed in imatinib-resistant GIST cell lines and recurrent GIST tissues.

RACK1 is a classical scaffold protein involved in the regulation of multiple signaling pathways and cellular functions. The individual WD40 repeats of RACK1 can simultaneously interact with different signaling molecules, allowing RACK1 to integrate inputs from distinct signaling pathways [14]. Previous research implied that RACK1 interacts with ABL, another major target of imatinib, only in transformed cells, and that the introduction of RAS enhances the association of RACK1 with ABL and subsequent alteration of signaling activities [29]. In this study, we found that imatinib-induced RACK1 expression reactivated the ERK and Akt signaling pathways in c-KIT-mutant GIST cells, thereby attenuating the antitumor effects of imatinib. Reactivation of ERK and Akt, the major downstream effectors of the c-KIT pathway, is one potential mechanism of resistance to anti-c-KIT therapies [30]. Imatinib treatment was ineffective in imatinib-resistant GIST cell lines in which reactivation of ERK and Akt, was not blocked, despite c-KIT inhibition. Increased RACK1 expression resulting from the inhibition of c-KIT activity in GIST cells contributed to imatinib resistance and consequently limited its efficacy. We also investigated the combination of imatinib and RACK1 shRNA in preventing GIST relapse *in vivo*. Combining RACK1 depletion with imatinib synergistically inhibited GIST growth and decreased relapsed tumor growth. This synergistic anti-proliferative effect was accompanied by an inhibition of ERK and Akt activation, which increases tumor cell apoptosis. Therefore, acquired imatinib resistance in GIST is at least partially driven by RACK1-mediated constitutive activation of the ERK and Akt pathways.

In summary, our data improve the understanding of how RACK1 regulates imatinib resistance in GIST and suggest potential inhibitory points that might be targeted to improve the efficacy of clinical therapies. Further study of the biological functions of RACK1 will be of great help in understanding mechanisms of GIST development and growth.

## MATERIALS AND METHODS

### Cell culture

The GIST882 cell line was kindly provided by Dr. Fletcher from Harvard Medical School. The GIST882 cell line is established from an untreated human GIST with a homozygous missense mutation in KIT exon 13, encoding a K642E mutant KIT protein. The GIST-T1 cell line was purchased from Cosmo Bio Co. LTD (Tokyo, Japan). The GIST-T1 cell line is derived from a metastatic plural tumor from the stomach of a Japanese woman and is characterized by a heterozygous deletion of 57 bases in KIT exon 11. GIST882 cells were cultured in RPMI-1640 (ATCC) supplemented with 15% FBS and 1% L-glutamine, and GIST-T1 cells were cultured in DMEM (ATCC) supplemented with 10% FBS. The identity of the cell lines was confirmed periodically throughout the study using SNP fingerprinting, and the KIT mutations were confirmed with RNA sequencing. Imatinib-resistant sublines of GIST-882 and GIST-T1 cells were obtained by culturing the cells in gradually increasing doses of imatinib. Cells that grew in 0.2, 0.4 and 1μM imatinib were obtained after 1, 2 and 4 months of culture with imatinib, respectively. The stability of the resistant phenotype was determined by culturing continuously in medium with corresponding concentrations of imatinib and assessing relative resistance for up to 5 months.

### Immunoblotting and immunoprecipitation

Protein extract was electrophoresed, transferred to polyvinylidene difluoride (PVDF) membranes, and incubated overnight with the indicated primary antibodies against RACK1 (Abcam; 1:600), phospho-c-kit (Santa Cruz Biotech; 1:800), c-kit (Santa Cruz Biotech; 1:600), myc (Abcam; 1:800), HA (Abcam; 1:800), phospho-ERK (Cell Signaling Tech; 1:1200), ERK (Cell Signaling Tech; 1:1000), phosphor-Akt (Cell Signaling Tech; 1:800), and Akt (Cell Signaling Tech; 1:1000). Membranes were then treated with the appropriate horseradish peroxidase (HRP)-conjugated secondary antibodies (Invitrogen). Detection was performed using the reagents provided in the ECLt Plus kit (GE Healthcare, Piscataway, NJ, USA).

For immunoprecipitation, cells were lysed with RIPA buffer [10 mM Tris-HCl (pH 7.5), 150 mM NaCl, 1% Nonidet P-40, 0.1% SDS, 1% sodium sarcosyl, 1 mM DTT] plus protease inhibitors. The soluble fraction was incubated overnight at 4°C with primary antibodies; protein G agarose beads (GIBCO-BRL) were then added and the solution was incubated for an additional hour at 4°C. Beads were washed three times with cold PBS or RIPA buffer, resuspended in SDS sample buffer, and boiled for 5 min. The eluted proteins were resolved on SDS-PAGE and immunoblot analysis was performed as described above. For His-tagged protein analysis, washed Ni-NTA agarose beads (Qiagen) were added to the cell lysates and incubated at 4°C for 4 hr. Eluted proteins were analyzed as above.

### Immunohistochemical assay

Briefly, slides were dehydrated in xylene and a graded alcohol series. Antigen retrieval was carried out with 0.01 M citrate buffer (pH 6.0) at 95°C for 10 min. Then slides were incubated with diluted primary antibodies against RACK1 (Abcam; 1:40), phospho-ERK (Cell Signaling Tech; 1:50), and phosphor-Akt (Cell Signaling Tech; 1:50) for 12 h, followed by incubations with biotinylated secondary antibody for 1 h, peroxidase-labeled streptavidin for 15 min (LSAB-2 System; DAKO, Glostrup, Denmark), and diaminobenzidine and hydrogen peroxide chromogen substrate plus diaminobenzidine enhancer (DAKO) for 10 min. Slides were counterstained with Mayer's hematoxylin. Known positive and negative control tissues were processed at the same time and under the same conditions.

### Cell viability assays

Cells were seeded at 5×10^3^ cells per well in 96-well plates. The next day, cells were rinsed and fresh medium was added with either DMSO or the various indicated reagents for 72 hr. Cell viability was assayed using a Cell Titer-Glo Luminescent Cell Viability Assay kit (Promega).

For colony formation assays, cells were plated into 10cm tissue culture dishes (5-10×10^6^ cells/dish) in a total volume of 10ml. After 24 hours, either DMSO or imatinib, RACK1 siRNA, or both reagents were added to the dishes at final concentrations of 1μM. At the end points of the assays, cells were fixed, stained with crystal violet, and photographed.

### RNA interference

siRNA pools against RACK1 and negative control were purchased from Dharmacon. Cells were plated into 6-well plates at 50% of confluence and transfected with Lipofectamine RNAiMAX (Invitrogen) per the manufacturer's instructions. Transfected cells were cultured for 96 hours and then treated with imatinib (1 μM) for further experiments.

### Xenograft studies

All procedures involving animals were reviewed and approved by the Institutional Animal Care and Use Committee at ZhongShan Hospital. For GIST-882 xenografts, 7- to 8-week old Balb/c nude mice were given right axillary subcutaneous injections of 5×10^6^ GIST-882 or GIST-882/RACK1 shRNA cells suspended in a 1:1 mixture of cold PBS and Matrigel in a total volume of 150μl. When tumors reached 150mm^3^, mice were randomized into four groups of twenty mice each. Mice were treated with vehicle or imatinib at 250 mg/kg twice daily for 4 days. Tumor volumes were determined with digital calipers using the formula (length×width×width)/2. Tumor weight was determined by weighing the wet tumor at the indicated time points.

## References

[1] Fletcher CD, Berman JJ, Corless C, Gorstein F, Lasota J, Longley BJ, Miettinen M, O'Leary TJ, Remotti H, Rubin BP, Shmookler B, Sobin LH, Weiss SW (2002). Diagnosis of gastrointestinal stromal tumors: a consensus approach. International journal of surgical pathology.

[2] Miettinen M, Lasota J (2001). Gastrointestinal stromal tumors--definition, clinical, histological, immunohistochemical, and molecular genetic features and differential diagnosis. Virchows Archiv.

[3] Hirota S, Isozaki K, Moriyama Y, Hashimoto K, Nishida T, Ishiguro S, Kawano K, Hanada M, Kurata A, Takeda M, Muhammad Tunio G, Matsuzawa Y, Kanakura Y (1998). Gain-of-function mutations of c-kit in human gastrointestinal stromal tumors. Science.

[4] Blanke CD, Demetri GD, von Mehren M, Heinrich MC, Eisenberg B, Fletcher JA, Corless CL, Fletcher CD, Roberts PJ, Heinz D, Wehre E, Nikolova Z, Joensuu H (2008). Long-term results from a randomized phase II trial of standard- versus higher-dose imatinib mesylate for patients with unresectable or metastatic gastrointestinal stromal tumors expressing KIT. Journal of clinical oncology.

[5] Blanke CD, Rankin C, Demetri GD, Ryan CW, von Mehren M, Benjamin RS, Raymond AK, Bramwell VH, Baker LH, Maki RG, Tanaka M, Hecht JR, Heinrich MC (2008). Phase III randomized, intergroup trial assessing imatinib mesylate at two dose levels in patients with unresectable or metastatic gastrointestinal stromal tumors expressing the kit receptor tyrosine kinase: S0033. Journal of clinical oncology.

[6] Demetri GD, von Mehren M, Blanke CD, Van den Abbeele AD, Eisenberg B, Roberts PJ, Heinrich MC, Tuveson DA, Singer S, Janicek M, Fletcher JA, Silverman SG, Silberman SL (2002). Efficacy and safety of imatinib mesylate in advanced gastrointestinal stromal tumors. The New England journal of medicine.

[7] Antonescu CR, Besmer P, Guo T, Arkun K, Hom G, Koryotowski B, Leversha MA, Jeffrey PD, Desantis D, Singer S, Brennan MF, Maki RG, Dematteo RP (2005). Acquired resistance to imatinib in gastrointestinal stromal tumor occurs through secondary gene mutation. Clinical cancer research.

[8] Debiec-Rychter M, Cools J, Dumez H, Sciot R, Stul M, Mentens N, Vranckx H, Wasag B, Prenen H, Roesel J, Hagemeijer A, Van Oosterom A, Marynen P (2005). Mechanisms of resistance to imatinib mesylate in gastrointestinal stromal tumors and activity of the PKC412 inhibitor against imatinib-resistant mutants. Gastroenterology.

[9] Heinrich MC, Corless CL, Blanke CD, Demetri GD, Joensuu H, Roberts PJ, Eisenberg BL, Von Mehren M, Fletcher CD, Sandau K, McDougall K, Ou WB, Chen CJ (2006). Molecular correlates of imatinib resistance in gastrointestinal stromal tumors. Journal of clinical oncology.

[10] Wardelmann E, Thomas N, Merkelbach-Bruse S, Pauls K, Speidel N, Buttner R, Bihl H, Leutner CC, Heinicke T, Hohenberger P (2005). Acquired resistance to imatinib in gastrointestinal stromal tumours caused by multiple KIT mutations. The lancet oncology.

[11] Javidi-Sharifi N, Traer E, Martinez J, Gupta A, Taguchi T, Dunlap J, Heinrich MC, Corless CL, Rubin BP, Druker BJ, Tyner JW (2015). Crosstalk between KIT and FGFR3 Promotes Gastrointestinal Stromal Tumor Cell Growth and Drug Resistance. Cancer research.

[12] Bauer S, Hartmann JT, de Wit M, Lang H, Grabellus F, Antoch G, Niebel W, Erhard J, Ebeling P, Zeth M, Taeger G, Seeber S, Flasshove M (2005). Resection of residual disease in patients with metastatic gastrointestinal stromal tumors responding to treatment with imatinib. International journal of cancer.

[13] Ron D, Chen CH, Caldwell J, Jamieson L, Orr E, Mochly-Rosen D (1994). Cloning of an intracellular receptor for protein kinase C: a homolog of the beta subunit of G proteins. Proc. Natl. Acad. Sci. USA.

[14] McCahill A, Warwicker J, Bolger GB, Houslay MD, Yarwood SJ (2002). The RACK1 scaffold protein: a dynamic cog in cell response mechanisms. Mol. Pharmacol.

[15] Berns H, Humar R, Hengerer B, Kiefer FN, Battegay EJ (2000). RACK1 is up-regulated in angiogenesis and human carcinomas. FASEB J.

[16] Wang Z, Zhang B, Jiang L, Zeng X, Chen Y, Feng X, Guo Y, Chen Q (2009). RACK1, an excellent predictor for poor clinical outcome in oral squamous carcinoma, similar to Ki67. Eur J Cancer.

[17] Nagashio R, Sato Y, Matsumoto T, Kageyama T, Satoh Y, Shinichiro R, Masuda N, Goshima N, Jiang SX, Okayasu I (2010). Expression of RACK1 is a novel biomarker in pulmonary adenocarcinomas. Lung Cancer.

[18] Cao XX, Xu JD, Liu XL, Xu JW, Wang WJ, Li QQ, Chen Q, Xu ZD, Liu XP (2010). RACK1: A superior independent predictor for poor clinical outcome in breast cancer. Int J Cancer.

[19] Myklebust LM, Akslen LA, Varhaug JE, Lillehaug JR (2011). Receptor for activated protein C kinase 1 (RACK1) is overexpressed in papillary thyroid carcinoma. Thyroid.

[20] Ruan Y, Sun L, Hao Y, Wang L, Xu J, Zhang W, Xie J, Guo L, Zhou L, Yun X, Zhu H, Shen A, Gu J (2012). Ribosomal RACK1 promotes chemoresistance and growth in human hepatocellular carcinoma. J Clin Invest.

[21] Wu M, Hemesath TJ, Takemoto CM, Horstmann MA, Wells AG, Price ER, Fisher DZ, Fisher DE (2000). c-Kit triggers dual phosphorylations, which couple activation and degradation of the essential melanocyte factor Mi. Genes Dev.

[22] Carver BS, Chapinski C, Wongvipat J, Hieronymus H, Chen Y, Chandarlapaty S, Arora VK, Le C, Koutcher J, Scher H, Scardino PT, Rosen N, Sawyers CL (2011). Reciprocal feedback regulation of PI3K and androgen receptor signaling in PTEN-deficient prostate cancer. Cancer cell.

[23] Chandarlapaty S, Sawai A, Scaltriti M, Rodrik-Outmezguine V, Grbovic-Huezo O, Serra V, Majumder PK, Baselga J, Rosen N (2011). AKT inhibition relieves feedback suppression of receptor tyrosine kinase expression and activity. Cancer cell.

[24] Corcoran RB, Ebi H, Turke AB, Coffee EM, Nishino M, Cogdill AP, Brown RD, Della Pelle P, Dias-Santagata D, Hung KE, Flaherty KT, Piris A, Wargo JA (2012). EGFR-mediated reactivation of MAPK signaling contributes to insensitivity of BRAF mutant colorectal cancers to RAF inhibition with vemurafenib. Cancer discovery.

[25] Lito P, Pratilas CA, Joseph EW, Tadi M, Halilovic E, Zubrowski M, Huang A, Wong WL, Callahan MK, Merghoub T, Wolchok JD, de Stanchina E, Chandarlapaty S (2012). Relief of profound feedback inhibition of mitogenic signaling by RAF inhibitors attenuates their activity in BRAFV600E melanomas. Cancer cell.

[26] Montero-Conde C, Ruiz-Llorente S, Dominguez JM, Knauf JA, Viale A, Sherman EJ, Ryder M, Ghossein RA, Rosen N, Faqin JA (2013). Relief of feedback inhibition of HER3 transcription by RAF and MEK inhibitors attenuates their antitumor effects in BRAF-mutant thyroid carcinomas. Cancer discovery.

[27] Miettinen M, Lasota J (2006). Gastrointestinal stromal tumors: review on morphology, molecular pathology, prognosis, and differential diagnosis. Arch Pathol Lab Med.

[28] Huss S, Künstlinger H, Wardelmann E, Kleine MA, Binot E, Merkelbach-Bruse S, Rüdiger T, Mittler J, Hartmann W, Büttner R, Schildhaus HU (2013). A subset of gastrointestinal stromal tumors previously regarded as wild-type tumors carries somatic activating mutations in KIT exon 8 (p. D419del). Modern Pathology.

[29] Huang CC, Liu CH, Chuang NN (2008). An enhanced association of RACK1 with Abl in cells transfected with oncogenic ras. Int J Biochem Cell Biol.

[30] Li F, Huynh H, Li X, Ruddy DA, Wang Y, Ong R, Chow P, Qiu S, Tam A, Rakiec DP, Schlegel R, Monahan JE, Huang A (2015). FGFR-Mediated Reactivation of MAPK Signaling Attenuates Antitumor Effects of Imatinib in Gastrointestinal Stromal Tumors. Cancer Discov.

